# How Older Adults Redefine Items on the PROMIS-57 Profile Patient-Reported Outcome Measure

**DOI:** 10.1093/geroni/igab046.2605

**Published:** 2021-12-17

**Authors:** Ann Gruber-Baldini, G Jay Unick, Veronica Fallon, Philip Heiser, Kimberly Bambarger, Lisa Shulman

**Affiliations:** 1 University of Maryland School of Meidcine, Baltimore, Maryland, United States; 2 University of Maryland, Baltimore, Baltimore, Maryland, United States; 3 University of Maryland School of Medicine, Baltimore, Maryland, United States

## Abstract

PROMIS® measures provide valid assessment of patient-reported outcomes, but have not been validated in older adults (especially aged 80+), including those with cognitive impairment. The objective of this project was to study how age-related role change effected the understanding of items on the PROMIS-57 Profile using cognitive interviews. Cognitive interviews were conducted with 38 adults, age 65+ with MoCA scores 10-30. Preliminary codes were created and then codes were added or modified as needed. Each interview was coded independently by two coders with differences resolved by consensus. The sample was 47% age 80+, 45% female, 18% African American, and 32% had a MoCA score between 10-17 (cognitively impaired). Thematic analysis of codes indicated that participants endorsed little or no impairment when they adapt to physical or cognitive disabilities by using economic means, instrumental support, physical aids, or by reducing activities. One respondent using grocery delivery services described no difficulty running errands or shopping. Another respondent reported no difficulty walking 15 minutes because they use a cane. Some reported no difficulty engaging in social roles when they restricted their activities due to disability or lack of appropriate social activities. Age-related changes effected responses on PROMIS-57 items. Findings suggest that age-related changes bias individuals to indicate less physical and cognitive impairment than their actual level of function. Physical functioning items show more bias for individuals with financial or instrument support, and social role items show more bias for those with restricted social networks.

